# Food Safety Attitudes in College Students: A Structural Equation Modeling Analysis of a Conceptual Model

**DOI:** 10.3390/nu5020328

**Published:** 2013-01-30

**Authors:** Rachelle Booth, Magaly Hernandez, Erica L. Baker, Tevni Grajales, Peter Pribis

**Affiliations:** 1 Department of Nutrition & Wellness, School of Health Professions, Andrews University, 8475 University Boulevard, Marsh Hall, Berrien Springs, MI 49104, USA; E-Mails: boothr@andrews.edu (R.B.); magaly@andrews.edu (M.H.); erica@andrews.edu (E.L.B.); 2 Department of Educational & Counseling Psychology, School of Education, Andrews University, 4195 Administration Drive, Bell Hall 159, Berrien Springs, MI 49104, USA; E-Mail: tevni@andrews.edu

**Keywords:** food safety, college students, attitudes

## Abstract

College students are one of the most at-risk population groups for food poisoning, due to risky food safety behaviors. Using the Likert Scale, undergraduate students were asked to participate in a Food Safety Survey which was completed by 499 students ages 18–25. Data was analyzed using SPSS and AMOS statistical software. Four conceptual definitions regarding food safety were defined as: general food safety, bacterial food safety, produce food safety, and politics associated with food safety. Knowledge seems to be an important factor in shaping students attitudes regarding general and bacterial safety. Ethnicity plays a role in how people view the politics of food safety, and the safety of organic foods.

## 1. Introduction

It is estimated that foodborne diseases cause approximately 76 million illnesses, 325,000 hospitalizations, and 5000 deaths annually in the United States [1]. College students are one of the most at-risk population groups due to risky food safety behaviors. Food safety is of particular concern in university settings because many college students are preparing meals for themselves and others for the first time in life [2]. Diarrhea is a major symptom of foodborne illness, however diarrhea in college students may also be attributed to other things such as excessive alcohol consumption, stress, anxiety, antibiotic use, and the use of food additives [3,4].

A study conducted at Ohio State University concluded that undergraduate students engage in behaviors that place them at risk, including risky food handling and food consumption and that college students are at a higher risk for foodborne diseases than the general population [2]. A cross-sectional online food safety survey found that young adults engage in risky eating behaviors like eating raw/undercooked foods of animal origin and other less then optimal safe food handling practices. Due to the challenges of obtaining a college education, many students eat whatever is convenient. Male respondents and whites consumed more risky foods compared with female respondents and nonwhites. Authors concluded that food safety educational efforts should focus on increasing knowledge particularly in males [5,6]. 

A study conducted at Kansas State University, examined the effect of educational intervention in food safety on college students. Findings indicated that interactive food safety education intervention resulted in improved food safety knowledge and beliefs. The strongest effects were seen in students who described that food safety principles were important to their future professions, e.g., health majors [7]. Students in health related majors had higher food safety knowledge scores than students in other disciplines, yet even they scored on average only 74% on a food safety knowledge test [4]. Dietetics and hospitality students seem to do better because their programs provide more hours of food safety education, and some require or offer food safety certifications [8,9]. A study conducted on four Japanese universities concluded that students who had more knowledge of food safety implemented more risk-reduction behaviors, as well as students who completed a basic food class or were working toward a degree in food or nutrition [10].

In developed societies food safety encompasses much more then just handling, preparation, and storage of food in ways that prevent foodborne illness. It embraces also concepts like attitudes toward environment (organic farming, vegetarian or vegan lifestyle), politics (regulation or deregulation of governmental food safety institutions), race, gender and other determinants. Although there have been several studies published on the many aspects of food safety among college students it is not clear what are the underlying factors associated with attitudes and beliefs toward food safety. The goal of this report was to: (i) test general nutritional knowledge among college students; (ii) to examine believes and attitudes toward food safety; and (iii) to report a theoretical model of the relationships between *General Food Safety*, *Bacterial Food Safety*, *Produce Safety*, and the *Politics of Food Safety*.

## 2. Experimental Section

### 2.1. Recruitment of Subjects

This cross-sectional observational study was done at Andrews University, which is a Seventh-day Adventist (SDA) institution of higher learning. SDAs represent a unique population to study due to their wide range of dietary habits. This religious group endorses a healthy lifestyle and recommends that members adhere to lacto-ovo-vegetarian diet. The study was approved by the University’s Institutional Review Board (IRB protocol # 11-143). Students from various undergraduate courses were asked to participate in the study. Participation in the study was voluntary. Data was collected in November of 2011. 

### 2.2. Assessment of Food Intake, Attitudes toward Food Safety, and Nutrition Knowledge

Each participant was asked to complete a four-page *Lifestyle Practices Survey*, which was comprised of four sections: 15 basic census questions (gender, age, ethnicity, marital status, class standing, questions regarding exercise habits, height, weight, vegetarian status), a 31-item Food Frequency Questionnaire (FFQ) to accurately ascertain the nutrition habits of the participants and their vegetarian status, a series of 27 statements regarding food safety using the Likert Scale ranging from 1 (strongly disagree) to 7 (strongly agree), and 18 true or false questions to test participants knowledge in general nutrition.

To assess the attitudes toward food safety we used a survey adapted from a study done by a doctoral student at Kansas State University [11]. Since our concept of food safety was broader we have added questions regarding environment and politics. The original survey contained 27 statements which were divided into four theoretical constructs: *General Food Safety*, *Bacterial Food Safety*, *Produce Food Safety*, and *Politics of Food Safety* (Supplementary). We performed principal complements analysis [12] to determine latent variables before testing the conceptual model. This analysis led to data reduction and elimination of seven variables (*Food Survey Questions* number 1, 5, 6, 9, 10, 20 and 21, see Supplementary), which did not contribute to any of the theoretical constructs. The total score for the different construct was computed by simply adding up the numerical value of the answers.

To assess the knowledge of the participants we added up their scores to the 18 true and false knowledge questions. We recoded the answers into 0 and 1 categories, and created grade categories using the following template: 90% to 100% correct *A*, 80% to 89% correct *B*, 70% to 79% correct *C*, 60% to 69% correct *D*, and 0 to 59% correct *F*.

### 2.3. Statistical Analysis

The data was analyzed using SPSS (version 18.0; SPSS, Inc., Chicago, IL, USA) for descriptive statistics (mean, standard deviation) and inferential statistics (ANOVA, *T*-test) and AMOS 7.0 statistical software for Structural Equation Modeling (SEM). SEM is a multivariate statistical method used in social sciences, and in health behavior research. SEM examines underlying relationships among variables in a conceptual model and helps to explain social or behavioral phenomena [13]. We performed descriptive statistics and measures of internal consistency on relevant variables before model testing and accounted for missing data. We tested two different models. Model comparisons were based on chi-square differences. Model modifications to determine the best-fit model were based on theoretical as well as statistical [Comparative Fit Index (CFI), Tucker-Lewis Index (TLI), Root Mean Square Error of Approximation (RMSEA)] judgments. *P* values less than or equal to 0.05 were considered statistically significant. 

## 3. Results

### 3.1. Sample Size and Characteristics

Overall, 550 participants completed the survey; 51 subjects were disqualified because they were not between the ages 18 to 25, leaving a study population of 499 (42% males and 58% females). The mean age was 20.0 years for males, and 19.8 years for females. The mean BMI was 24.3 for males and 23.5 for females. The majority of males and females were SDAs, Caucasian, omnivore and would be considered knowledgeable about nutrition (Table 1).

**Table 1:** Selected Characteristics of Participants.

	Males	Females
Gender (%, *n*)	42 (209)	58 (290)
Age (years; mean, SD)	20 (±1.7)	19.8 (±2.1)
BMI (kg/m^2^; mean, SD)	24.3 (±4.9)	23.5 (±5.2)
Seventh-Day Adventist (%, *n*)	94 (197)	93 (269)
Ethnicity (%, *n*)		
Caucasian	38 (72)	30 (79)
African American	27 (50)	28 (19)
Hispanic	16 (31)	23 (74)
Asian	15 (29)	7 (59)
Other	15 (7)	12 (32)
Knowledge (%, *n*)		
A (90%–100%)	4 (8)	7 (21)
B (80%–89%)	31 (64)	27 (78)
C (70%–79%)	35 (74)	38 (110)
D (60%–69%)	29 (61)	26 (74)
F (0–59%)	1 (2)	2 (7)
Vegetarian Status (%, *n*)		
Vegetarian	31 (65)	37 (107)
Omnivore	69 (144)	63 (183)
BMI stands for Body Mass Index		

### 3.2. Food Safety Attitudes

Food safety was divided into four underlying constructs: *General Food Safety*, *Bacterial Food Safety*, *Produce Safety*, and *the Politics of Food Safety*. Ethnicity, vegetarian status, knowledge, and gender were tested for significant differences in attitudes toward these four constructs.

#### 3.2.1. General Food Safety

**Figure 1:**
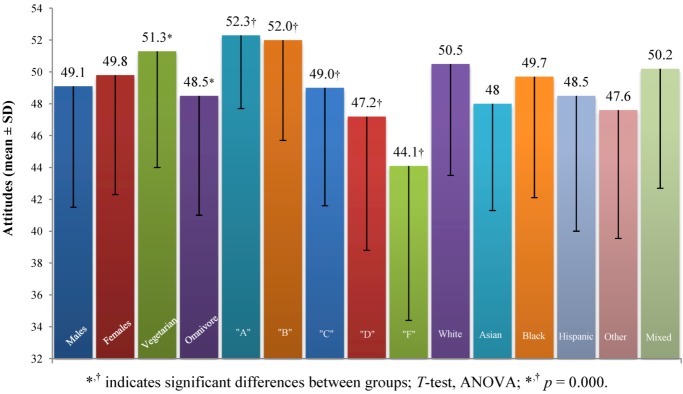
Attitudes toward *General Food Safety* for selected groups.

There were nine questions assessing *General Food Safety* construct, (*Food Survey Questions* number 12, 13, 17, 18, 22, 24, 25, 26 and 27, see Supplementary—Lifestyle Survey) which was measured on an inverse scale (the questions were negatively phrased), meaning a high score indicated a negative attitude towards general food safety, however we have recoded the answers converting the *General Food Safety* into a positive construct (range 9–63; values below 36 indicate negative attitudes, values above 36 indicate positive attitudes toward general food safety). There were significant differences regarding general food safety between vegetarians (51.3 ± 7.3) and omnivores (48.5 ± 7.5), with vegetarians having a more positive view towards general food safety (*p* = 0.000). There were also significant differences between the five groups divided according to the level of knowledge (ANOVA, *F* (4, 494) = 9.1, *p* = 0.000). *Post-hoc* comparison using the LSD test indicated that the following groups were significantly different from each other: “A” (52.3 ± 4.6) from “C” (49.0 ± 7.4) and “D” (47.2 ± 8.4) and “F” (44.1 ± 9.7); “B” (52.0 ± 6.3) from “C” and “D” and “F”; “C” from “D”. As knowledge increased the students tended to have a more positive attitude towards general food safety (Figure 1). 

#### 3.2.2. Bacterial Food Safety

**Figure 2:**
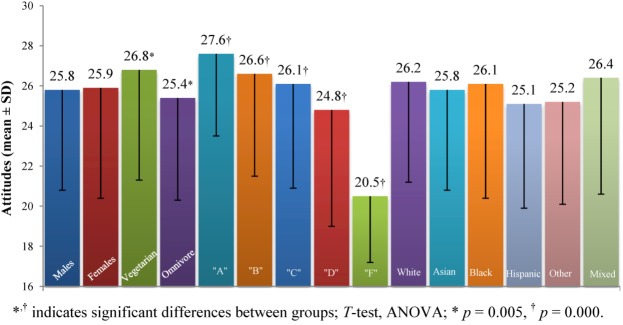
Attitudes toward *Bacterial Food Safety* for selected groups.

The *Bacterial Food Safety* construct consisted of five questions (*Food Survey Questions* number 11, 14, 15, 16, and 23; range 5–35; values above 20 indicate positive attitudes, values below 20 indicate negative attitudes toward bacterial food safety). This construct was measured on a direct scale, high scores indicate a positive attitude toward bacterial food safety; low scores indicate a negative attitude toward bacterial food safety (Figure 2). There was a significant difference between vegetarians (26.8 ± 5.6) and omnivores (25.4 ± 5.1), with vegetarians having a more positive view towards bacterial safety than omnivores (*p* = 0.005). We observed significant differences in the five groups divided according to level of knowledge [ANOVA, *F* (4, 494) = 5.2, *p* = 0.000]. *Post-hoc* comparison using the LSD test indicated that the following groups were significantly different from each other: “A” (27.6 ± 4.1) from “D” (24.8 ± 5.8) and “F” (20.5 ± 3.4); “B” (26.6 ± 5.1) from “D” and “F”; “C” (26.1 ± 5.2) from “D” and “F”; “D” from “C”; and “F” from “D”. As knowledge decreased, positive attitudes decreased as well.

#### 3.2.3. Produce Safety

**Figure 3:**
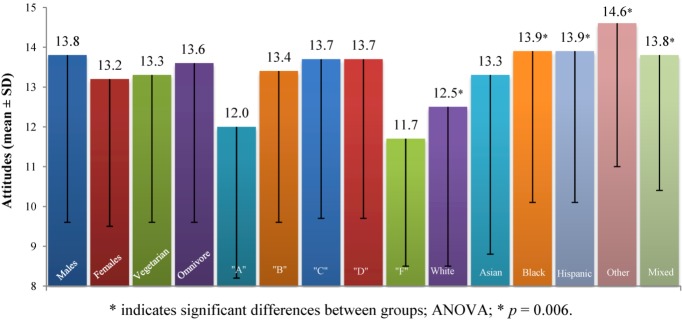
Attitudes toward *Produce Safety* for selected groups.

The *Produce Safety* construct consisted of three questions (*Food Survey Questions* number 2, 3, and 4; range 3–21; values above 12 indicate positive attitudes, values below 12 indicate negative attitudes toward produce safety). It assessed attitudes toward organic foods, use of pesticides and traditional farming practices. A higher score indicates a more positive view towards produce safety (Figure 3). We observed significant differences in the six groups divided according to race (ANOVA, *F* (5, 493) = 3.3, *p* = 0.006). *Post-hoc* comparison using the LSD test indicated that the following groups were significantly different from each other: Caucasians (12.4 ± 4.0) from African Americans (13.9 ± 3.8), Hispanics (13.9 ± 3.7), participants of other race (14.6 ± 3.6), and participants of mixed racial background (13.8 ± 3.4).

#### 3.2.4. The Politics of Food Safety

**Figure 4:**
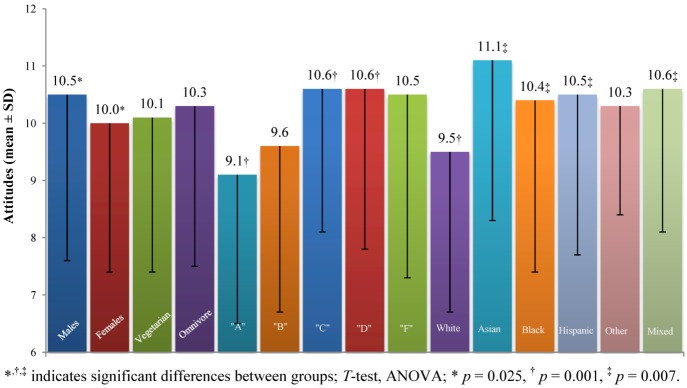
Attitudes toward *Politics of Food Safety* for selected groups.

*The Politics of Food Safety* construct involved three questions (*Food Survey Questions* number 7, 8, and 19; range 3–21; values above 12 indicate positive attitudes, values below 12 indicate negative attitudes toward the politics of food safety). It assessed attitudes toward food safety regulations including views the two major political parties in US embrace toward this issue. A higher score indicates a more positive view towards politics of food safety (Figure 4). There was a significant difference between genders, with males (10.5 ± 2.9) having a slightly more positive view towards politics of food safety than females (10.0 ± 2.6; *p* = 0.025). We observed significant differences in the five groups divided according to level of knowledge (ANOVA, *F* (4, 494) = 4.5, *p* = 0.001). *Post-hoc* comparison using the LSD test indicated that the following groups were significantly different from each other: “A” (9.1 ± 2.6) from “C” (10.6 ± 2.6) and “D” (10.6 ± 2.8); and “B” (9.6 ± 2.9) from “C” and “D”. Knowledge played an inverse role, with positive attitudes somewhat increasing as the knowledge scores decreased. We observed significant differences in the six groups divided according to race (ANOVA, *F* (5, 493) = 3.2, *p* = 0.007). *Post-hoc* comparison using the LSD test indicated that the following groups were significantly different from each other: Caucasians (9.5 ± 2.8) from Asians (11.1 ± 2.8), African Americans (10.4 ± 3.0), Hispanics (10.5 ± 2.8), and participants of mixed racial background (10.6 ± 2.5).

### 3.3. Attitudes toward Hand Washing

**Figure 5:**
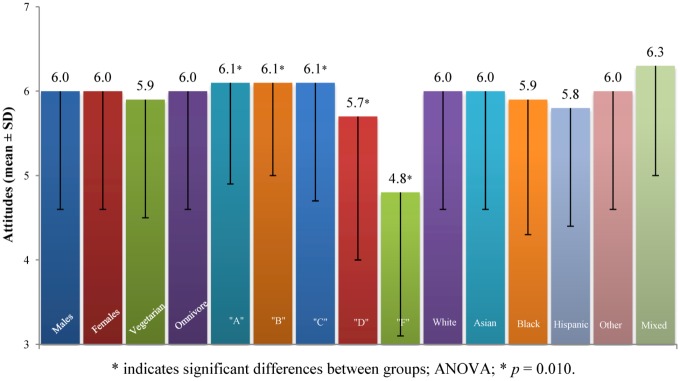
Attitudes toward hand washing for selected groups.

According to Center for Disease Control and Prevention, keeping hands clean through improved hand hygiene is one of the most important steps we can take to avoid getting sick and spreading germs to others [14]. Our survey incorporated one question (*Food Survey Question* number 11, range 1–7, values above 3.5 indicate positive attitude toward hand washing, values below 3.5 indicate negative attitude toward hand washing) testing the attitude of college students toward hand washing. This question was part of the *Bacterial Food Safety* construct, however given the importance of this practice we are reporting the data separately. There were no significant differences between the genders, vegetarian status or race (Figure 5). However, we observed significant differences in the five groups divided according to level of knowledge (ANOVA, *F* (4, 494) = 3.3, *p* = 0.010). *Post-hoc* comparison using the LSD test indicated that the following groups were significantly different from each other: “A” (6.1 ± 1.2) from “F” (4.8 ± 1.7); “B” (6.1 ± 1.1) from “D” (5.7 ± 1.6) and “F”; “C” (6.1 ± 1.4) from “D” and “F”. As knowledge decreased the negative attitudes increased.

### 3.4. Theoretical Model of the Relationship between General Food Safety, Bacterial Food Safety, Produce Safety, and the Politics of Food Safety

This study examined the underlying concepts that college students have towards food safety using the SEM statistical method. SEM is a powerful multivariate statistical method being used in social sciences, and with increasing frequency in health behavior research [15]. SEM examines the underlying relationships among variables in the model and helps to explain social or behavioral phenomena. Our model was constituted by four sets of concepts: *General Food Safety, Bacterial Food Safety, Produce Safety,* and *Politics of Food Safety*.

The hypothesized model was assessed by AMOS version 7.0 using the maximum likelihood method. The model was evaluated by four fit measures: (**a**), the chi square (**b**), the Comparative Fit Index (CFI) (**c**), the Good-of-Fit-Index and (**d**), the Root Mean Square of approximation (RMSEA). The results for three out of the four indices support the proposed model. The chi-square had a value of 318.457 (Df = 163, *n* = 499), *p* = 0.000, indicating a non-acceptable match between the proposed model and the observed data. However, due to the size of the sample, additional fitted indices were considered. The CFI = 0.915, GFI = 0.941, both of them indicating an excellent fit of the model. The RMSEA measures the discrepancy between sample coefficients and the population coefficients equals 0.044 (confidence interval 0.037–0.051) indicating an acceptable fitting. 

**Figure 6:**
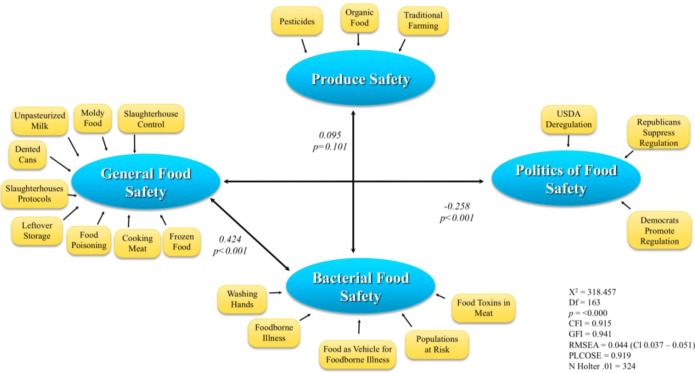
Structural Equation Modeling.

Findings support a model (Figure 6) that suggests that there is a direct medium correlation between *Bacterial Food Safety* and *General Food Safety* (*r* = 0.424, *p* < 0.001). When positive attitudes toward general food safety are high, attitudes toward bacterial safety are positive as well. The more likely people were to engage in dangerous food practices, such as eating food from dented cans and drinking unpasteurized milk, the less likely they were to practice bacterial safety, such as washing hands. There is an indirect medium correlation between *General Food Safety* and *Politics of Food Safety* (*r* = −0.258, *p* < 0.001). As negative attitudes toward *General Food Safety* increased, positive attitudes toward the *Politics of Food Safety*, or the legislation of food safety increased as well. This could be an indicator that people who have a negative attitude towards general food safety are more inclined to shift responsibility to the government to watch and monitor food safety, instead of doing it themselves. There is a marginal direct weak correlation between *Bacterial Food Safety* and *Produce Safety* (*r* = 0.095, *p* = 0.101).

### 3.5. Limitations of Study

Several potential limitations of the study should be considered. This is an observational study which included both genders, and an age group of 18 to 25. This study was conducted on the campus of an American private university. A large sample size was studied, but some groups might be underrepresented. Knowledge, attitudes, intentions, and self-reported practices do not always correspond to behaviors [16]. Although SEM is a sophisticated analytical tool for testing theoretical models in behavioral or social science, the analyses are correlational which makes it difficult to establish causality. Because of the isolation of variables in the model are impossible, all models must be looked at as only an estimation of reality. 

## 4. Discussion

The intent of this study was to test general nutritional knowledge among college students, examine believes and attitudes toward food safety and test and define a theoretical model. The survey has shown that the level of basic nutritional knowledge including food safety on our campus is insufficient and lacking, only 34.2% of students demonstrated adequate knowledge (A or B grades), with 63.9% showing serious deficiencies (C and D grade) and 1.8% failing (F grade). There are significant differences in attitudes toward food safety in college students across several groups. Students who are vegetarian and have better knowledge of nutrition tend to have more positive attitudes toward general and bacterial food safety. Ethnicity plays a role in attitudes towards organic foods and produce safety, with participants of other race, having the most positive attitude toward this construct. Asians had the most positive attitude towards politics of food safety. 

We have tested four different constructs: *General Food Safety*, *Bacterial Food Safety*, *Produce Safety*, and *the Politics of Food Safety*. All the constructs, with the exception of the *Politics of Food Safety* produced positive attitudes. In *General Food Safety*, *Bacterial Food Safety*, *Produce Safety* as nutritional knowledge increased the attitudes became more positive. Nevertheless, the surprising finding was the inverse relationship between knowledge and attitudes toward politics of food safety, the less the students knew about nutrition the more positive was their attitude toward politics of food safety. This relates to our observation from the SEM model that students with negative attitudes toward general food safety seem to view legislation and food safety regulation positive. Further research with more detailed models is necessary to confirm our findings.

Washing hands with soap and water is the most convenient and efficient way of removing pathogens from hands [17]. Our data clearly shows, that with decreasing general knowledge, including food safety knowledge (there were four food safety questions in the *Food review* survey, questions number 2, 12, 16, and 17) in nutrition, there was a significant rise in negative attitudes toward this simple food safety practice. Previous research has shown that knowledge does not always correspond with improved food safety behaviors [7,16], however we could assume that the more negative attitudes students have toward hand washing, the less likely they will wash their hands after using the toilet or before, during and after preparing food. Food safety education, as part of general food education or separately, should be encouraged because it leads to more positive attitudes toward hand hygiene.

In our population approximately one third of the participants embraced vegetarian diet because the study was done on a campus of a SDA university which actively endorses this lifestyle. Vegetarians tend to consume more produce (fruits and vegetables). It was unexpected that there were not significant differences between vegetarians and omnivore regarding attitudes toward organic foods, use of pesticides, and produce safety. Possible explanation could be that our study population was made up from young college students. In our previous research we have observed that younger people seem to be more motivated to embrace vegetarian lifestyle for moral (it is wrong to kill animals) or environmental (vegetarian lifestyle is much more protective for the environment) reasons while middle aged people seem to be more motivated by health (vegetarians live longer and are less sick) reasons [18].

To our knowledge, the present study has been first to apply sophisticated multivariate statistical methods to study the underlying concepts that college students might have toward food safety. The model confirms that those who hold negative attitudes toward general food safety and possibly practice risky food safety behaviors are not very progressive with bacterial safety and hand washing as well. In both of these constructs (*General Food Safety* and *Bacterial Food Safety*) we observed significant differences in attitudes according to the level of knowledge (higher knowledge means more positive attitudes) stressing the importance of educational interventions.

Additional research is needed to better understand issues in food safety and to study broader concepts of food safety among students.

## 5. Conclusions

Knowledge seems to be an important factor in shaping student’s attitudes regarding general and bacterial safety. Ethnicity plays a role in how people view the politics of food safety and perceive the safety of organic foods. Better nutritional knowledge leads to more positive attitudes toward hand washing. Nutrition education and food safety education should be encouraged.

## Supplementary Files

Supplementary File 1 Supplementary File (PDF, 785 KB)
