# Supplementary material for: Food Safety Attitudes in College Students: A Structural Equation Modeling Analysis of a Conceptual Model
Source: Nutrients. 2013 Jan 30;5(2):328–39. doi: 10.3390/nu5020328 (PMC3635197; doi:10.3390/nu5020328)

Lifestyle survey

For each of the following questions, please mark an ☒ in the one box or circle the number which best describes your answer.

INFORMED CONSENT FORM

TITLE: Attitudes towards Food Safety in College Students

PURPOSE OF THE STUDY: To ascertain the attitude of college students in a Seventh-day Adventist university towards food safety.

INCLUSION CRITERIA: In order to participate, I recognize that I must be an Andrews University student who is 18 years old or older of sound mind that is currently enrolled in classes as of Fall 2011.

RISKS AND DISCOMFORTS: I have been informed that there are no physical or emotional risks to my involvement in this study.

BENEFITS/RESULTS: I accept that I will receive no remuneration for my participation, but that by participating, I will help the researcher to understand the attitudes towards food safety in a college aged population. I understand I will earn 25 extra points for participating in this study.

VOLUNTARY PARTICIPATION: I understand that my involvement in this survey is voluntary and that I may withdraw my participation at any time without any pressure, embarrassment, or negative impact on me. I also understand that my participation is confidential and will never be disclosed.

CONTACT INFORMATION: In the even that I have any questions or concerns with regard to my participation in this research project, I understand that I may contact the researcher, Rachelle Booth, at boothr@andrews.edu, or 269-471-8243 or Dr. Peter Pribis at pribis@andrews.edu or 269-471-3386.

☐ I agree with the Informed Consent Form

☐ I do not agree with the Informed Consent Form

Census questions

**What is your gender:**	1☐ Male	2☐ Female
**How old are you:**	__________ (years)	
**What is your ethnicity:**	1☐ White, non-Hispanic	2☐ Oriental
	3☐ Black (African American)	4☐ American Indian
	5☐ Hispanic	6☐ Other
	7☐ Mixed racial background	
**What is your marital status:**	1☐ Never married	2☐ First time married
	3☐ Separated	3☐ Divorced
	5☐ Widowed	
**What is your class standing:**	1☐ Freshman	2☐ Sophomore
	3☐ Junior	4☐ Senior
	5☐ Graduate	
**How many meals do you eat daily:**	1☐ one	2☐ two
	3☐ three	4☐ four
	5☐ five	6☐ more than five
**How many hours on average do you sleep every night?**		__________ (hours)
**How many days a week do you engage in aerobic physical activity at least 30 minutes or longer?**		________ (number of days)
**How many days a week do you engage in muscle-strengthening physical activity?**		________ (number of days)
**How many days a week do you engage in stretching physical activity?**		________ (number of days)
**Are you a Seventh-day Adventist?**	1☐ Yes	2☐ No
**What is your height:**	__________________ (ft)	__________________(in)
**What is your weight:**	__________________ (lb)	
**What lifestyle are you following:**	1☐ **Non-vegetarian** (eat meat, dairy products, eggs, fish, vegetables, fruits, legumes, grains)<br>2☐ **Pesco-vegetarian** (eat fish, vegetables, fruits, legumes, grains)<br>3☐ **Lacto-ovo-vegetarian** (eat dairy products, eggs, vegetables, fruits, legumes, grains)<br>3☐ **Vegan** (eat vegetables, fruits, legumes, grains)	
**How long are following this lifestyle?**	**……………………….. (years)**	

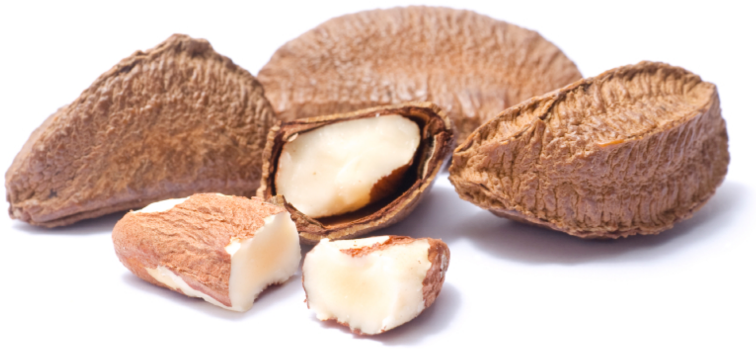

Food Frequency Questionnaire

Code	1	2	3	4	5	6	7
**Food**	Never or rarely	1-3 per month	1 per week	2-4 per week	5-6 per week	1 per day	2+ per day
Beef (hamburger, ground beef, meatballs, casserole, etc.)							
Chicken or turkey (roasted, stewed, broiled, fried, in casserole, etc.)							
Pork (bacon, sausage, ham, chops, ribs)							
Lamb (steak, roast, stew, sausage, salami, etc.)							
Fish (tuna, salmon, herring, trout, mackerel et.)							
Milk (whole, 2%, skim)							
Cheese (American, cheddar, mozzarella etc.)							
Yogurt (regular, low fat)							
Sour cream (whole or low fat)							
Egg (fried, boiled, scrambled, egg salad etc.)							
Fresh fruit (apples, bananas, pears, grapes, oranges, etc.)							
Canned fruits (peaches, pineapple, pears, etc.)							
Dried fruits (dates, figs, raisins)							
Salads (iceberg, romaine)							
Raw vegetables (tomatoes, carrots, onions, etc.)							
Cooked vegetables (broccoli, cauliflower, peas, yams, etc.)							
Legumes (lentils, beans, soybeans)							
Regular bread or pasta (made from white flour)							
Whole grain bread or pasta							
Whole grain cereals (granola, shredded wheat, etc.)							
Flax seeds (ground flax seed or flax seeds oil)							
Nuts (walnuts, almonds, peanuts, peanut butter, cashews etc.)							
Drinking water (including sparkling)							
Soft drinks and coffee (Coke, Pepsi, Mountain Dew etc.)							
Caffeine free beverages (decaf soft drinks, decaf coffee, etc.)							
Juices (orange, grape, apple etc.)							
Tofu (scrambled, silken, etc.)							
Meat substitutes (Vegi-Franks, Fri-Chick, Veja Links, etc.)							
Soy or rice drinks (Silk, Rice dream or homemade)							
Pizza (Pizza Hut, Dominos, etc.)							
Fast food (hamburgers, burritos, fried chicken)							

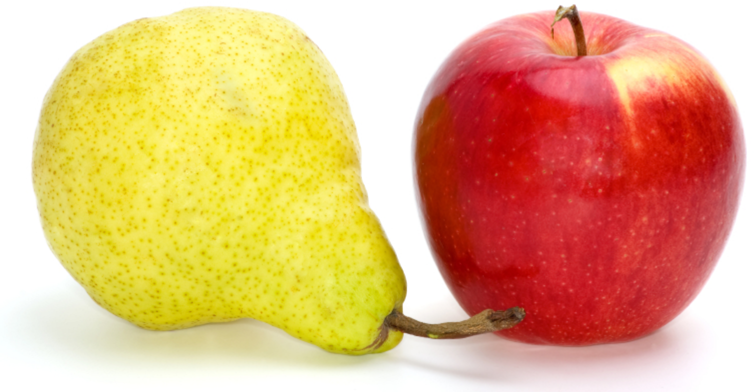

Food Survey Questions

		Strongly Disagree			Agree			Strongly Agree
1	The food supply in the USA is generally safe	1	2	3	4	5	6	7
2	Eating produce treated with pesticides makes food dangerous to health	1	2	3	4	5	6	7
3	Organic food is healthier because it is higher in minerals and vitamins.	1	2	3	4	5	6	7
4	Organically farmed food is always healthier choice than food from traditional farming practices	1	2	3	4	5	6	7
5	It is better to buy traditionally farmed produce that is flawless than organic produce that is not.	1	2	3	4	5	6	7
6	Food additives are safe because the government regulates them	1	2	3	4	5	6	7
7	Republicans try to suppress food safety regulations	1	2	3	4	5	6	7
8	Democrats tend to pass more legislation regarding the food industry than Republicans	1	2	3	4	5	6	7
9	The food supply is safer today than it was in the past	1	2	3	4	5	6	7
10	It is important to sanitize sponges and cutting boards with chlorine bleach solution	1	2	3	4	5	6	7
11	It is important to wash hands with hot water and soap before eating or working with food	1	2	3	4	5	6	7
12	Drinking unpasteurized milk is safe	1	2	3	4	5	6	7
13	Buying dented cans is safe	1	2	3	4	5	6	7
14	Any food can be a vehicle for foodborne illness	1	2	3	4	5	6	7
15	Foodborne toxins are most likely to affect young children, pregnant women, and the elderly.	1	2	3	4	5	6	7
16	Meat is more susceptible to food toxins than fruits and vegetables	1	2	3	4	5	6	7
17	Each slaughterhouse controls the microbial contamination of the meat	1	2	3	4	5	6	7
18	It is fine to eat food with mold as long as the mold has been cut off.	1	2	3	4	5	6	7
19	Foodborne illnesses are increasing due to USDA deregulation	1	2	3	4	5	6	7
20	Foodborne illnesses can be passed from person to person	1	2	3	4	5	6	7
21	The US needs more regulation regarding food safety	1	2	3	4	5	6	7
22	It is safe for inspectors at slaughterhouses rely solely on their sense of sight, smell, and touch to examine animals for signs of disease	1	2	3	4	5	6	7
23	All foods can be carriers for foodborne illness	1	2	3	4	5	6	7
24	Leaving leftovers out after a meal is safe, as long as the food is reheated before eating again	1	2	3	4	5	6	7
25	Food poisoning only kills people who are already sick	1	2	3	4	5	6	7
26	As long as the pink color has been cooked out of the meat, it is safe to eat	1	2	3	4	5	6	7
27	Freezing food kills all the bacteria, making it safe to eat	1	2	3	4	5	6	7

Food Review

	Code	1	2
1	Eating a diet high in whole grains, legumes, fruits, and vegetables makes people healthier	T	F
2	Eating cans that are bulging with air is safe	T	F
3	Oranges and lemons are a good source of vitamin C	T	F
4	Eating junk food increases risk for chronic disease	T	F
5	Vitamin E is called the sunshine vitamin	T	F
6	Vitamin B12 is produced by bacteria	T	F
7	A vegetarian lifestyle is the healthiest diet option	T	F
8	It is impossible to get all nutrients from a vegan diet without supplementation	T	F
9	Soluble fiber is able to lower cholesterol	T	F
10	The building block of proteins is fatty acids	T	F
11	The sweetest type of sugar is glucose	T	F
12	Beef is usually contaminated with listeria	T	F
13	People need to eat meat because they cannot get enough protein from plant sources.	T	F
14	Fruits and vegetables should be washed in cold running water before eating	T	F
15	Eating saturated fat increases cholesterol levels	T	F
16	Meat should be heated to at least 160 degrees to kill foodborne pathogens.	T	F
17	The danger zone where bacteria multiples rapidly is between 41 and 140 degrees Fahrenheit	T	F
18	Milk and dairy products are the only source of calcium and vitamin D	T	F

Thank you for participating in this survey

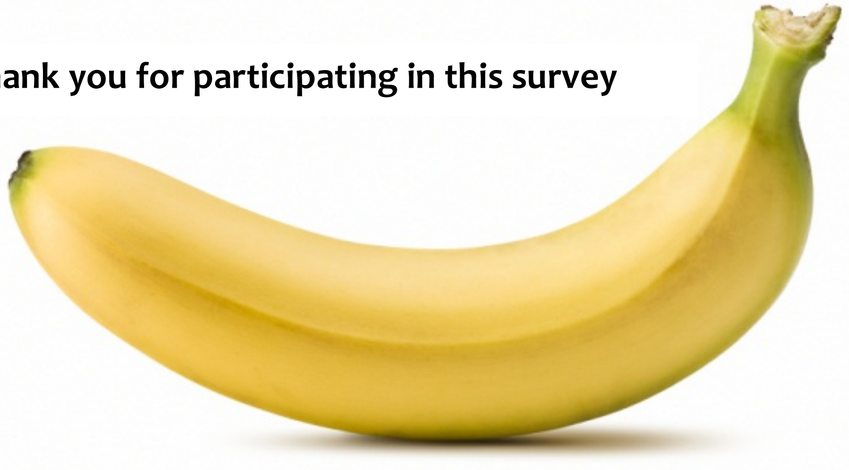

Supplement: Supplementary File 1 — Supplementary File (PDF, 785 KB) [file nutrients-05-00328-s001.pdf]
